# Sleep Apnea Detection Using Wavelet Scattering Transformation and Random Forest Classifier

**DOI:** 10.3390/e25030399

**Published:** 2023-02-22

**Authors:** Ahmed I. Sharaf

**Affiliations:** Deanship of Scientific Research, Umm Al-Qura University, Mecca 24382, Saudi Arabia; aisharaf@uqu.edu.sa

**Keywords:** Obstructive Sleep Apnea, wavelet scattering transformation, entropy-based features, random forest, ECG signals processing, automatic detection

## Abstract

Obstructive Sleep Apnea (OSA) is a common sleep-breathing disorder that highly reduces the quality of human life. The most powerful method for the detection and classification of sleep apnea is the Polysomnogram. However, this method is time-consuming and cost-inefficient. Therefore, several methods focus on using electrocardiogram (ECG) signals to detect sleep apnea. This paper proposed a novel automated approach to detect and classify apneic events from single-lead ECG signals. Wavelet Scattering Transformation (WST) was applied to the ECG signals to decompose the signal into smaller segments. Then, a set of features, including higher-order statistics and entropy-based features, was extracted from the WST coefficients to formulate a search space. The obtained features were fed to a random forest classifier to classify the ECG segments. The experiment was validated using the 10-fold and hold-out cross-validation methods, which resulted in an accuracy of 91.65% and 90.35%, respectively. The findings were compared with different classifiers to show the significance of the proposed approach. The proposed approach achieved better performance measures than most of the existing methodologies.

## 1. Introduction

OSA is a common sleep breathing disorder caused by a decrease in airflow during sleep (Hypopnea) or by a complete cessation of airflow (Apnea) due to an upper respiratory illness [1]. PSG is a widely used diagnostic tool to study sleep disorders. The test is conducted in the sleep clinic while the patient is sent to sleep with many electrodes placed on various body parts to record multiple physiological activities. This procedure involves connecting the patient to the PSG equipment and collecting biological signals overnight. A physician evaluates and diagnoses these signals under the established recommendations of the American Academy of Sleep Medicine (AASM). Unfortunately, this approach causes inconvenience to the patients due to the lengthy testing intervals. In addition, the increased cost of the lab setup is another difficulty in using this approach [2].

Various methods have been applied to study OSA, such as snoring sound analysis [3], pulse oximetry [4,5,6], and ECG signal [7]. Since sleep apnea disorder significantly affects heart rate, cardiovascular activity, and other related ECG characteristics, the ECG can capture these variations and then quantify the disrupted breath during the occurrence of the apnea. Moreover, the ECG provides an inexpensive and non-invasive alternative to the PSG. Authors and researchers have used a single-lead ECG signal, which also contains relevant information on the heart activity affected by the OSA. When an apnea event occurs, a drop in the heart rate is commonly observed, followed by a rise before the end of the event [8]. These apneic events will change the ECG signal frequency amplitude for a certain period. Therefore, analyzing the ECG signals for the classification and detection of OSA has gained much attention from several researchers [6,9].

A time-domain feature extraction within each RR peak to the peak time interval and SVM classifier was developed to distinguish the sleep apnea [10]. A framework was proposed based on the analysis of ECG signals to detect hidden patterns using correlation of the cyclical variations of the heart rate curve [11]. A spectral-domain analysis was combined with the reconstructed phase space [12]. A set of spectral and non-linear features was extracted from the raw ECG signals. Then, an SVM classifier was applied using the Gaussian radial kernel. The RR interval parameters were also used as the input features to classify and detect the OSA utilizing a set of ELM classifiers [13]. A different approach was developed using complex wavelet transform employed statistical features. Then, the classification is performed using logistic boosting [14]. A set of non-linear features were extracted from the wavelet coefficients obtained from ECG signals, followed by a features selection method. Then, an SVM classifier with RBF kernel was applied to categorize the ECG segments [15].

Radar technology is a non-contact detection approach that processes the transmitted and reflected radio signals to monitor the vital signs and chest movements of the patient [16]. It can detect respiration efforts at a long distance, even in the presence of material between the radar and the subject. This method could obtain significant results when integrated with other techniques, such as the deep learning method, to increase its robustness [17]. Various investigations used deep learning methods such as CNN, RNN, LSTM, and GRU to find hidden patterns, improving the diagnostics and detection of OSA [9,18,19,20]. However, this model requires a large number of training samples to provide satisfactory results, which increases the computational cost of the training phase. The settings of the deep learning model usually require various experiments to determine the optimal values of the parameters.

The WST is an effective tool for analyzing nonstationary signals, which provides time and frequency resolutions, stability to deformations, and conserves high-frequency information. The WST solved some limitations of the wavelet transform, such as translation invariance. The WST simulates some characteristics of deep learning, such as multiscale contractions, linearization of hierarchical symmetries, and sparse representation [21]. Therefore, the WST has the advantages of the conventional and deep learning approaches with low computational costs. It has achieved significant performance in audio recognition, and signal classifications [22,23].

This work presented an enhanced approach to detect and classify sleep apnea events from single-lead ECG signals using wavelet scattering transformation and random forest classifier. Similar to the Wavelet Transformation, the WST is used for signal decomposition, decreasing the signal computational cost by dividing the signal into smaller sub-bands. Moreover, the WST can perform similar to a CNN because it has cascaded decomposition and convolution operations, which propose it an effective preprocessing method for classification problems. A set of high-order and entropy-based measures was used to formulate the feature space to improve the detection and classification of the OSA. Different classifiers were used to test and benchmark the performance of the proposed method. These classifiers were compared with the random forest classifier to obtain the best result for the experiment. The experiment was repeated with many feature selection techniques to reduce the original feature space.

The remainder of this paper is organized as follows. The details of the dataset and the proposed approach are explained in Section 2. The experiment setup and the obtained findings are presented in Section 3. In Section 4, the discussion of the obtained results is presented, and finally, Section 5 contains the conclusion.

## 2. Materials and Methods

**Figure 1:**
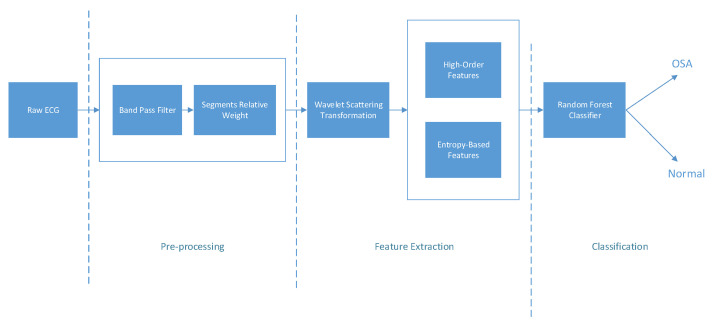
Block diagram of the proposed approach.

The proposed method consisted of three main phases, as shown in Figure 1. The first phase included the band-pass filter and noisy segment detection. The raw ECG signals were divided into 60 s per segment. In the second phase, the scattering wavelet transformation was applied to filtered segments using two filter banks to obtain the scattering coefficients. The scattering coefficients were then mapped to 2D instead of 3D. Then, a set of high-order statistics and entropy-based features was extracted. A sequential feature selection was used to reduce the original features into a compact group. A PCA dimension reduction was also applied to determine the best reduction method. Then, the obtained compact set was fed to the random forest classifier to detect and classify the OSA.

### 2.1. Dataset Description

This paper used the Physionet Apnea-ECG dataset for training, testing, and benchmarking [24]. The ECG signals were collected at 100 Hz, with 16 bits resolution, and the lead V2 electrode configuration was adjusted using an overnight PSG recording. The records varied in length from 7 to 10 h. According to the American Academy of Sleep Medicine guidelines, an expert affixed (minute-by-minute) apnea labels to the PSG signals. A minute of data is considered an apnea if at least one apnea or hypopnea event occurs during the minute. The dataset was categorized into three main groups: apnea (A), borderline (B), and normal (C), according to the value of the AHI as follows:

- Normal: AHI <5.
- Mild: 5≤ AHI <15.
- Moderate: 15≤ AHI <30.
- Severe: AHI ≥30.

**Figure 2:**
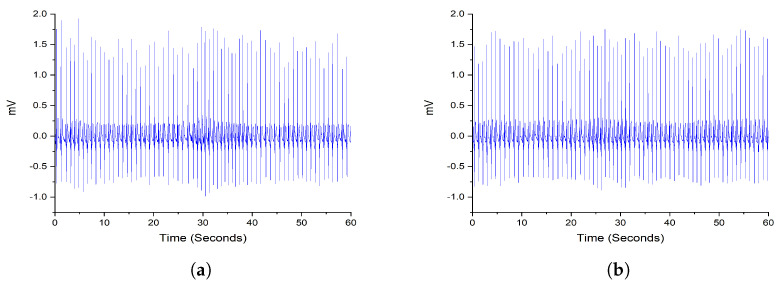
An example of Apnea and normal ECG signals acquired randomly from the dataset for 1 min duration. (**a**) Apnea ECG. (**b**) Normal ECG.

There are 70 ECG recordings in the dataset divided into training and testing subsets with 35 subjects per class. The training subset includes 10,454 annotated segments as normal and 6511 segments annotated as apneic collected from 35 subjects. Figure 2 shows a random sample from the dataset to illustrate the difference between the healthy and apneic classes.

### 2.2. Preprocessing

**Figure 3:**
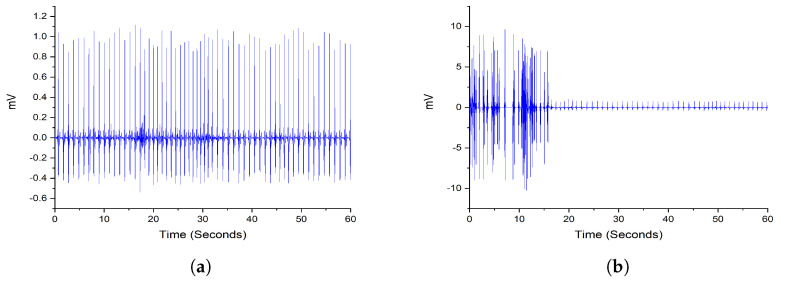
An example of noisy and clean data segments from a random patient. (**a**) A clean sample ECG segment with a weight of 0.98. (**b**) A noisy sample ECG segment with a weight of 0.79.

The raw ECG signals are divided into 60 s intervals per patient, which proved to be the most effective interval durations [25]. The first step in the preprocessing phase is to apply a finite impulse response bandpass filter with cut-off frequencies from 3 to 45 Hz to remove the power line interference. After using the bandpass filter, the relative weight between each data segment for each patient was computed to identify the significance of each data segment and reduce noisy data. The ACF was calculated between any two segments. Then, the cosine similarity was applied between the obtained vectors to measure the similarity between the data segments as follows:

(1) $$w_{st}=\frac{\left(X_{s}-{\bar{X}}_{s}\right){\left(X_{t}-{\bar{X}}_{t}\right)}^{T}}{\sqrt{\left(X_{s}-{\bar{X}}_{s}\right){\left(X_{s}-{\bar{X}}_{s}\right)}^{T}}\sqrt{\left(X_{t}-{\bar{X}}_{t}\right){\left(X_{t}-{\bar{X}}_{t}\right)}^{T}}}$$

where $w_{st}$ is the weight between two data segments *s* and *t*, $X_{s}$ and $X_{t}$ are the ACFs vectors obtained from the segments and ${\bar{X}}_{s}$, ${\bar{X}}_{t}$ are the mean values of $X_{s}$ and $X_{t}$, respectively. After applying Equation (1), it generates a symmetrical matrix containing the similarities of each data segment for the patient. Then, the weight of each segment was obtained by calculating the average score of this segment. A threshold value λ defines the clean and noisy segments. When the segment is classified as noisy, it will be dropped from the computation. An example of the difference between noisy and clean segments with a 1-min interval for a random patient is shown in Figure 3. In this paper, the value of λ is set to 0.8, as suggested by [15].

### 2.3. Wavelet Decomposition Using Scattering Wavelet

The WST is an improved time-frequency analysis method based on wavelet transformation. The WST produces a stable translation-invariant and informative signal representation. The WST is very effective for classification problems because of its stability to deformations and preserves class discriminability. It consists of cascaded decomposition and convolution operations performed on the input signal [26]. Let the function f(t) be the signal under study. Then, a low-pass filter ϕ(t) and a wavelet mother function ψ are constructed to cover the signal’s frequencies. The low-pass filter provides local translation-invariant descriptions of the input signal at a threshold scale of *T*. The family of wavelet indices is denoted by $\Lambda_{k}$ with a corresponding octave frequency resolution of $Q_{k}$. The high pass filter banks are constructed by dilating the wavelet function over the input signal, which are denoted by $\psi_{jk}$ for each $j,k\in \Lambda_{k}$. The WST is calculated using a convolutional network that iterates over the traditional wavelet transformation. The convolved signal oscillates at a scale of $2^{j}$, and averaging such signal equals zero. A nonlinear (modulus/rectifier) operator was used for the convolved segment to eliminate such oscillations. The conventional operator $S_{0}\left(f\left(t\right)\right)=f*\phi_{J}\left(t\right)$ produces the local invariant translation features of the input function *f*. However, the high-frequency information of the input signal was lost because of the convolution operation. Therefore, the modulus transformation was applied to recover the lost data as follows:

(2) $$\left|\omega_{1}\right|f={\left\{S_{0}f\left(t\right),\left|f*\psi_{j_{1}}\left(t\right)\right|\right\}}_{j_{1}\in \Lambda_{1}}$$

The first order scattering coefficients are obtained by using the average of the modulus coefficient with $\phi_{J}$ as follows:

(3) $$S_{1}f\left(t\right)={\left\{\left|f*\psi_{j_{1}}\right|*\phi_{J}\left(t\right)\right\}}_{j_{1}\in \Lambda_{1}}$$

The information lost during the average process could be retrieved by extracting the high-frequency coefficients of $f\left(t\right)*\psi_{j_{1}}$ as follows:

(4) $$\left|\omega_{2}\right|\left|f*\psi_{j_{1}}\right|={\left\{S_{1}f\left(t\right),||f*\psi_{j_{1}}\left|*\psi_{j_{2}}\left(t\right)\right|\right\}}_{j_{2}\in \Lambda_{2}}$$

Then, the second-order scattering coefficients are obtained similar to Equation (3):

(5) $$S_{2}f\left(t\right)={\left\{||f*\psi_{j_{1}}\left|*\psi_{j_{2}}\right|*\phi_{J}\left(t\right)\right\}}_{j_{i}\in \Lambda_{i}},i=1,2$$

By generalizing, the wavelet modules convolutions are obtained as follows:

(6) $$U_{m}f\left(t\right)={\left\{|||f*\psi_{j_{1}}|*\ldots |*\psi_{j_{m}}|\right\}}_{j_{i}\in \Lambda_{i}},i=1,2,\cdots ,m$$

The higher-order scattering coefficients are calculated by the average of $U_{m}f\left(t\right)$ with $\phi_{J}$ to obtain $S_{m}f\left(t\right)$ as follows:

(7) $$S_{m}f\left(t\right)={\left\{|||f*\psi_{j_{1}}|*\ldots |*\psi_{j_{m}}|*\phi_{J}\left(t\right)\right\}}_{j_{i}\in \Lambda_{i}},i=1,2,\cdots ,m$$

**Figure 4:**
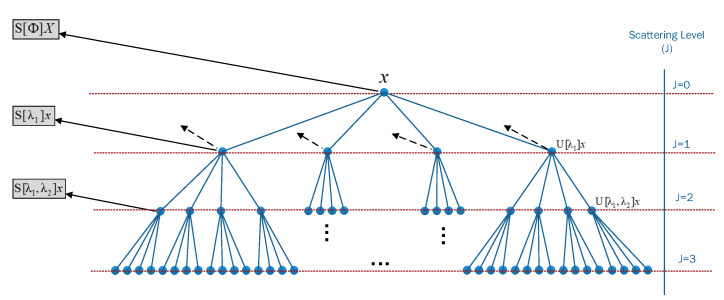
The hierarchy view of the wavelet scattering network.

The final form of scattering coefficients could be rewritten in a vector form as follows:

(8) $$Sf\left(t\right)={\left\{S_{m}f\left(t\right)\right\}}_{0\leq m\leq l}$$

where $S_{m}$ is the aggregates scattering coefficient of all orders to describe the features of the input signal, and *l* is the maximum level of decomposition. Figure 4 shows an illustration of the steps required to calculate the WST coefficients at various levels of decomposition, and outcome features are obtained as an aggeration of these coefficients.

As the number of layers increases, the energy of scattering coefficients diminishes, while the first two levels contain 99% of the total power of the input signal [27]. Another investigation utilized WST to extract characteristics from ECG and found that the optimal value of scattering layers is $l^{2}$ [28]. The obtained scattering features Sf(t) are stable to local deformation as an inherited property from the wavelet transformation. The scattering decomposition can capture slight variations in the amplitude and duration of the ECG signals. Therefore, the wavelet scattering tree was employed to generate a robust representation of ECG signals that minimize disparities. In this study, the characteristics of ECG signals were extracted using a second-order scattering network to reduce the computational complexity.

### 2.4. High-Order and Entropy-Based Features

#### 2.4.1. High-Order Moments Features

Higher-order moments are valuable for processing non-Gaussian and non-stationary signals; the variations among these features could improve the classification. The statistical mean, standard deviation, *skewness*, and *kurtosis* are defined as follows:

(9) $$\mu_{x}=\frac{1}{N}\sum_{i=1}^{N}x(n)$$

(10) $$std={\left(\frac{1}{N}\sum_{i=1}^{N}{\left(x\left(n\right)-\mu_{x}\right)}^{2}\right)}^{1/2}$$

(11) $$skewness=\frac{\frac{1}{N}\sum_{i=1}^{N}{\left(x\left(n\right)-\mu_{x}\right)}^{3}}{{\left(\frac{1}{N}\sum_{i=1}^{N}{\left(x\left(n\right)-\mu_{x}\right)}^{2}\right)}^{3/2}}$$

(12) $$kurtosis=\frac{\frac{1}{N}\sum_{i=1}^{N}{\left(x\left(n\right)-\mu_{x}\right)}^{4}}{{\left(\frac{1}{N}\sum_{i=1}^{N}{\left(x\left(n\right)-\mu_{x}\right)}^{2}\right)}^{2}}$$

Higher-order moments are essential in investigating non-Gaussian and non-stationary signals whose divergences are neither systematic nor predictable.

#### 2.4.2. Shannon Entropy

Shannon Entropy is a fundamental measure of the complexity and uncertainty of any state. The concept of the Shannon entropy is the foundation concept of the information theory, which is computed as follows:

(13) $$H\left(x_{i}\right)=-\sum_{i\in N}p\left(x_{i}\right)\log p(x_{i})$$

where $x_{i}$ is the instance variable, $P(x_{i})$ is the probability of the variable, and *N* is the length of the variables. However, the Shannon entropy suffers from the following limitations:

- The patterns in the time-series data must be complex enough to model the data. Therefore, it requires a lot of data to populate all histogram bins to obtain a dense histogram.
- The calculation of the Shannon entropy is a time-consuming process.

#### 2.4.3. Approximate Entropy

ApEn is a time-domain measure used to solve short data length and complex signal problems. It is also used for the quantization of physiological signal irregularity. The ApEn is a robust measure used to determine the HRV irregularity of the sleep apnea signals to perform detection and classification [29]. For a given u(i), *N* are the input signal and its corresponding length. The ApEn is determined as follows: let x(i) is a random segment where x(i)∈u(i), and *m* is the length of the segment such that: x(i)=[u(i),u(i+1),…,u(i+m−1)]. Then, the distance between two segments x(i),x(j) is determined as follows:

(14) $$d\left[x\left(i\right),x\left(j\right)\right]=max{\left|[u(i+k-1)-u(j+k-1)]\right|}_{\left\{k=1,2,\cdots ,m\right\}}$$

Then, let $C_{i}^{m}\left(r\right),\forall i=\left\{1,2,\cdots ,N-m\right\}$ is defined as:

(15) $$C_{i}^{m}\left(r\right)=\frac{\left|d[x(i),x(j)]\leq r\right|}{N-m+1}$$

where *r* is a similarity tolerance threshold defined as r=0.25∗STD, and STD is the standard deviation of the data segment. The higher the value of *r*, the more information may be lost. When the value of *r* is low, the sensitivity to noise increases significantly. Then, the average randomness for the whole segments is calculated as follows:

(16) $$\Phi^{m}\left(r\right)=\frac{1}{N-m+1}\sum_{i=1}^{N-m+1}\ln\left(C_{i}^{m}\left(r\right)\right)$$

Finally, by generalization of Equation (16), the value of the ApEn is calculated as:

(17) $$ApEn\left(m,r,N\right)=\Phi^{m}\left(r\right)-\Phi^{m+1}\left(r\right)$$

#### 2.4.4. Sample Entropy

The SampEn is a complexity measure based on the concept of the ApEn. However, it is defined as the negative value of the logarithm of the conditional probability between two segments of data [29]. In addition, the Sample Entropy has two advantages when compared to the ApEn, which are:

(1) The value of the ApEn depends on the value of *r*, which does not guarantee consistency.
(2) The value of the ApEn depends on the length of the data segment.

The distance between two vectors is determined as shown in Equation (14). The SampEn is calculated as follows:

(18) $$C_{j}^{m}\left(r\right)=\frac{\left|d[x(i),x(j)\leq r\right|}{N-m},\forall i\neq j$$

where *m* is the value of the embedded dimension that determines the number of samples contained in each vector.

(19) $$SampEn\left(m,r,N\right)=-\ln\frac{C_{j}^{m+1}\left(r\right)}{C_{j}^{m}\left(r\right)}$$

#### 2.4.5. Spectral Entropy

SpEn is a complexity measure used to obtain the uncertainty and randomness of the signal’s power spectrum [30]. The signal is converted to its corresponding power spectrum density, then, after normalization, the Shannon entropy is computed as follows:

(20) $$SpEn\left(x\right)=\frac{-\sum P_{k}\log P_{k}}{\log\left(N\right)}$$

where $\sum P_{k}=1$ is the probability distribution, and *N* is the total number of frequencies.

#### 2.4.6. Attention Entropy

AttEn is a novel complexity measure focusing on discovering key observations (local maxima and local minima) from the input data series [31]. The main advantages of this measure are its linear time complexity and robust performance. Moreover, it can detect different patterns in the time series, which solves the Shannon entropy’s limitations. Instead of counting the frequency of all observations, the AttEn evaluates the frequency distribution of the intervals between the most significant observations in a time series. The calculation of the AttEn for an input signal x(t) is performed as follows: if a data point $x_{i}$ belongs to the key pattern, then the interval between this key point and the previous key points $x_{j}$ is calculated as f(i,j)=x(i)−x(j). Then, the count of the interval values is stored in a variable *F*. Then, the Shannon entropy is computed over the frequency distribution of the intervals *F* using Equation (13).

#### 2.4.7. Cumulative Residual Entropy

The CRE is an alternative measure to the Shannon entropy, which retains some of its properties and overcomes some limitations of the Shannon entropy [32]. Instead of using the density function as Shannon entropy, it uses the cumulative distribution function. The CRE is calculated as follows:

(21) $$CRE\left(X\right)=-\sum_{i\in N}p\left(\left|X\right|>x_{i}\right)\log p(\left|X\right|>x_{i})$$

where *N* is the length of the signal *X*.

### 2.5. OSA Classification

**Figure 5:**
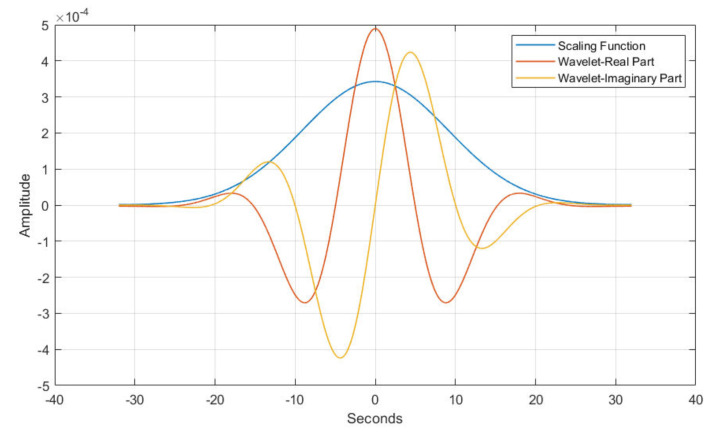
The low filter with 60 s invariance scaled function.

**Figure 6:**
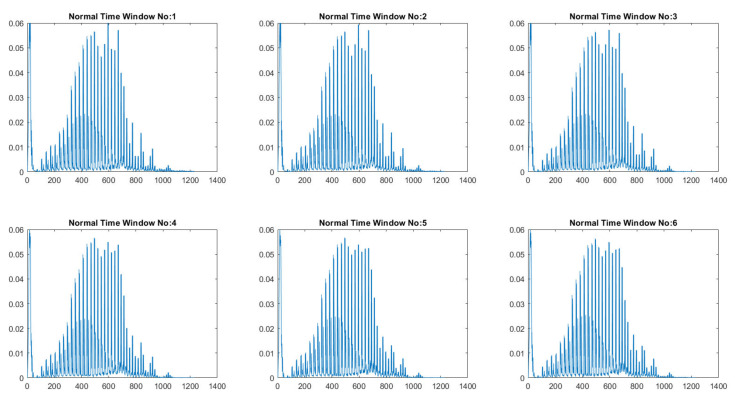
Scattering coefficients obtained from a healthy case.

**Figure 7:**
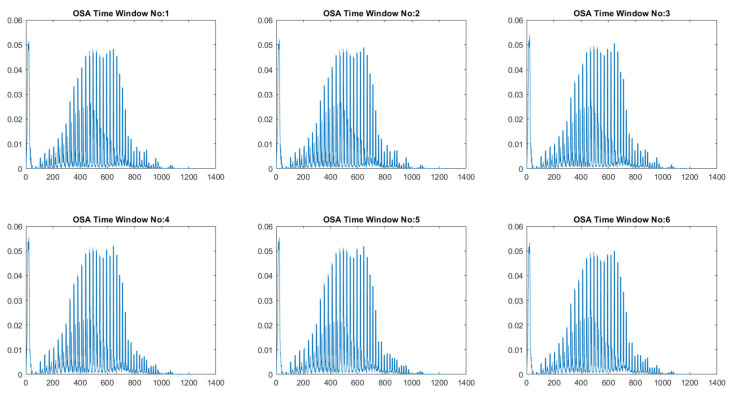
Scattering coefficients obtained from an OSA case.

**Figure 8:**
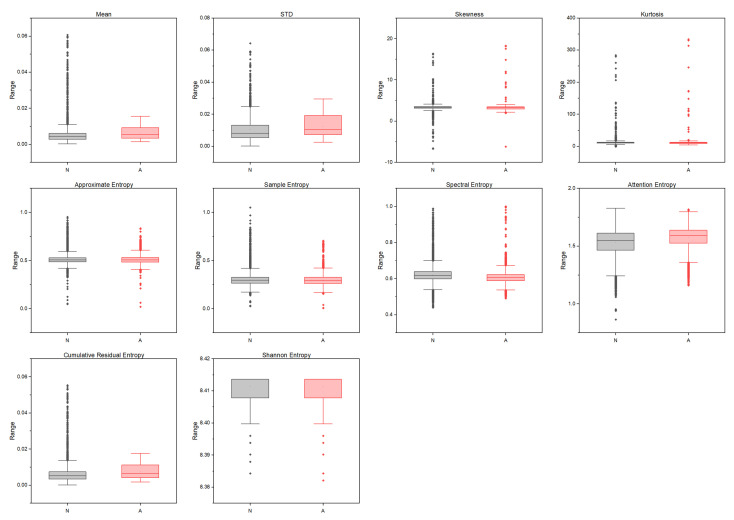
A box-whisker plot illustrating the feature space (without normalization) where *N* and *A* represent the Normal (gray) and Apnea (red) cases, respectively.

Ensemble learning is a robust approach that combines the predictions of various simple low-accuracy models instead of searching for a complicated high-accuracy learning model. The weak learner’s main advantage is the training’s fast speed and the less complexity in predicting initial predictions. RF is a bootstrap aggregation method that creates multiple replicas of the training samples. Each sample’s clone is used to train the weak classifier. Thus, integrating multiple uncorrelated decision tree models improves prediction accuracy. The dataset contains 10,454 normal and 6511 apnea ECG segments of 1-min duration extracted from 35 annotated recordings used for this experiment. Then, the weight calculation was performed according to Equation (1), which detected 120 noisy segments. Then, the Gabor wavelet function was used to perform the wavelet decomposition, and the invariance scale was set to 60 s with a sampling frequency of 100 Hz. The grid search obtained the best values of the scattering network layers as Q1=8 and Q2=1 wavelet per octave. Figure 5 shows the used Gabor wavelet function and its low-pass filter $\phi_{J}\left(t\right)$. The output of the constructed scattering wavelet is a 3D tensor with dimensions of [1227×6×489]. The scattering coefficients were then reshaped to [2934×1227] to fit the classifier’s input, where each column and row represents a scattering path and a time window. Since there are six windows, the total number of samples is [6×489]. The difference between the healthy and OSA scattering coefficients is presented in Figure 6 and Figure 7, respectively. Then, the features described in Section 2.4 were extracted from these coefficients to represent the feature vectors. Figure 8 shows the box-whisker plot of each feature obtained from WST coefficients to illustrate its significance. The proposed method includes two steps of classification to ensure its effectiveness and efficiency. The values of each feature were normalized using the z-score before forwarding to the classifier as formulated below:

(22) $$Z_{i}=\frac{x_{i}-\bar{X}}{\sigma (X)}$$

where $x_{i}$ represents the value of the feature vector *X* at the *i*-th location. $\bar{X}$ and σ(X) represent the mean and standard deviation of the same feature vector *X*, respectively. The first step performs classification and detection of each segment with a 1-min duration on the entire training dataset using 10-fold cross-validation. In this step, the complete set of features was used for the training and validation. In the second step, the classifier is performed on a subject-by-subject 1-min duration using the Hold-out selection method. In addition, different classifiers are used to compare the results with the RF and hence find the best classifier. The used classifiers are as follows: AdaBoost, ExtraTree, GaussianNB, KNN, LDA, LR, QDA, RF, SGD, SVM, and XgBoost.

## 3. Results

The proposed method used the Physionet dataset for validation and testing as mentioned in Section 2.1, which contains 16,965 ECG segments gathered from 35 subjects with a time duration of 1 min. These segments were annotated by a domain expert to distinguish the normal (healthy) and the apnea segments. A total of 120 noisy segments were detected from the weight calculation mentioned in Section 2.2 using a threshold of 0.8. Then, the WST was applied to the cleaned segments only for signal decomposition. A set of high orders and entropy-based features were extracted from the obtained scattering coefficients as mentioned in Section 2.4. Then, the dataset was given to a set of classifiers to perform the experiment and obtain the best results. The details of the experiment results and selection methods are given below.

### 3.1. Performance Measures

The performance of the proposed method is based on the analysis of the confusion matrix and additional statistical measures. The confusion matrix obtained the main terms of the classification performance TP, TN, FP, and FN. Using these measures: ACC, SEN, SPE, Precision, F1 score, ROC, and the Area under ROC are obtained. In this work, the ACC describes the total number of correctly classified ECG segments from both the apnea and normal conditions. Sensitivity describes the correctly identified apnea segments from the entire apnea segments, and specificity describes the correctly classified normal cases from the total normal segments. Precision represents the ratio of positive prediction. In case of conflict between sensitivity and precision, the F1 measure was used to measure the harmonic mean between them. The AUC measures the separation between the classification categories.

### 3.2. Experimental Results

The RF classifier was applied to the feature space extracted from the WST coefficients of the 1-min ECG signals to distinguish the normal and apnea segments. Additionally, the performance of the proposed method was studied using different classifier sets to compare their performance and obtain the best classifier. For example, Naive Bayes classifier, linear models such as LR and SGD, functional models such as SVM, LDA and QDA, lazy classifiers such as KNN, and ensemble methods such as RF, ExtraTrees, AdaBoost, and XGBoost are all used to benchmark the performance of the proposed method. Two cross-validations methods allow various testing and validation with existing methods, namely, hold-out and k-fold. The hold-out procedure is performed by partitioning the data set into 50% for training and 50% for testing. The k-fold approach divides the dataset into consecutive (k−1) partitions and the remaining one for testing. The experiment was repeated ten times for each cross-validation method to obtain the average performance measure. Table 1 shows the experimental results using the hold-out method using different classifiers and performance measures which are written as (mean ± standard deviation) formula. It is observed from this table that the RF achieved the best average accuracy of 90.23%, a specificity of 91.99%, and a precision of 87.24%, which is higher than the ExtraTrees with 0.24%. The F1 score shows that ExtraTrees are better than the RF with a small amount. This experiment proved the effectiveness of RF and ExtraTrees when compared to other techniques. The experiment has also been performed using 10-fold cross-validation to validate its results with different classifiers, as shown in Table 2. It is observed from this table that the RF achieved the best accuracy of 91.5 and higher deviation than the ExtraTrees, which is the best classification algorithm for this problem. The RF also achieved the best results in the Precision measure. The ExtraTrees achieve the best F1 result with a slight deviation compared to RF.

**Figure 9:**
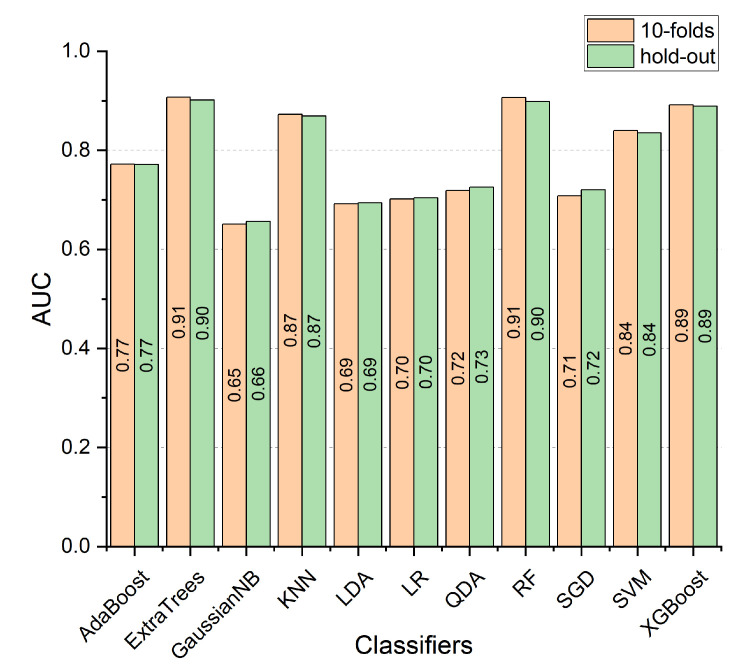
The area under the curve (AUC) is illustrated using 10-fold and hold-out cross-validation methods without PCA.

Moreover, Cohen’s kappa coefficient was used to examine the agreement between categorical variables *X* and *Y*. For example, kappa can be used to compare the ability of various estimators to classify subjects into one of several classes. Cohen’s kappa coefficient was performed on each classifier with hold-out and 10-fold cross-validation. The experiment was repeated ten times to obtain the average results. The RF and ExtraTrees outperformed the other classifiers with the same kappa value of 0.81 using the 10-fold method. Then, the XgBoost, KNN, and SVM placed the second level of agreement with kappa values of 0.78, 0.74, and 0.68, respectively. The remaining classifiers are in the lowest level of agreement with kappa values less than or equal to 0.5. The same results were obtained by repeating this procedure using the hold-out method, which indicates that the RF and ExtraTrees are the best classifiers. The AUC was used to evaluate the performance of different classifiers by computing the area under the ROC. As shown in Figure 9, the RF and ExtraTrees outperformed the remaining classifiers with a value of 0.91. Then, the XgBoost, KNN, and SVM achieved second place with values of 0.89, 0.87, and 0.84, respectively. The GaussianNB achieved the worst result with a value of 0.65. The PCA is a robust dimension reduction approach used to eliminate the feature space. The PCA was applied to the hold-out and 10-fold experiments to study the effect of dimensions reduction, as shown in Table 3 and Table 4, respectively. From Table 3, the RF achieved the best results with an average accuracy of 86.83%, then ExtraTrees scored 86.54%. The KNN, XgBoost, and SVM results are 86.07%, 85.68, and 82.68%, respectively. Then, the AdaBoost, LR, SGD, and LDA achieved 73.16%, 72.33%, 72.08%, and 71.23%, respectively. Finally, the QDA and GaussianNB obtained 69.45% and 69.01%, respectively. It is observed from Table 4 that RF remains the best classifier. However, the overall accuracy was decreased to 88% with a low standard deviation value. Then, the ExtraTrees, KNN, XgBoost, and SVM scored 87.38%, 87.04%, 86.71%, and 84.05%, respectively. Then, the remaining classifiers achieved relative accuracy closer to 72.5%. The results show that the RF performs significantly better than the other classifier. Then, an SFS was implemented to reduce the feature space and obtain a compact subset of features using the RF regressor. The forward selection method reduced the feature space to 5 attributes instead of 10: spectral entropy, standard deviation, kurtosis, approximate entropy, and the mean. The significance of the spectral entropy and standard deviation equals 0.2. Then, the kurtosis, approximate entropy, and mean scored 0.13, 0.1, and 0.1, respectively. The usage of WST could reduce the computational cost in the training phase as the time complexity of the scattering wavelet could be computed as $O(L\times Nlog_{2}\left(N\right))$, where *N* is the length of the signal, and *L* is the number of decomposition levels.

## 4. Discussion

The results presented in the previous section demonstrated the details of the experiments and concluded that the ensemble RF classifier was the most significant. The WST was applied to the raw ECG signals using two filter banks with values of *Q*1 = 8 and *Q*2 = 4 obtained by the search grid. The scattering wavelet outperformed various transformation methods because of its behavior based on aggregation and maximization of features. This behavior is similar to the CNN operation. However, it reduces the time-consuming and processing time when compared with CNNs. The extracted features are shown in Figure 8 were obtained from the scattering wavelet coefficient to produce the search space. These features show the significance of classifying OSA apnea and healthy subjects through various techniques. The RF outperformed the other classifiers with significant outcomes using k-fold and hold-out cross-validation methods. Although, the difference between the k-fold and hold-out cross-validation was not substantial. The k-fold method improved the results of the hold-out with an average accuracy, sensitivity, specificity, precision, and F1 equal to 1.3%, 1.12%, 0.32%, 0.54%, and 0.91%, respectively. Cohen’s kappa was performed on the full feature set with the k-fold and hold-out methods, and the most significant results were obtained for the RF and ExtraTrees classifiers. The higher value of Cohen’s kappa measure indicates a higher agreement between the classifiers. Then, the AUC was calculated for the same experiment and produced the same significance for the RF and ExtraTrees. Furthermore, two feature reduction approaches were applied to validate the proposed method’s performance using PCA and SFS. Firstly, PCA was applied to summarize the feature space into fewer dimensions using geometric projection. PCA is a powerful data reduction method when the hidden patterns increase the variance of the projections onto orthogonal components. However, the results obtained by the PCA are less significant when compared with the original feature space. The RF scored an average accuracy equal to 88% and 86.83% for the k-fold and the hold-out methods, respectively. Secondly, the SFS was applied to the feature space using the bi-directional method to select the most valuable features from the original features set. The SFS obtained spectral entropy, standard deviation, kurtosis, approximate entropy, and the mean as the most significant features.

### Comparative Analysis

The proposed method achieved an average accuracy of 91.65% in the minute-by-minute duration using 10-fold cross-validation, as shown in Table 2. The proposed approach provided an effective solution to detect and classify the OSA regarding the low quality of some recordings. Moreover, the feature extraction did not depend on any extracted parameters from the ECG signals, reducing the probability of incorrect classification. Another advantage of the proposed approach is its low computational cost, making it suitable for integrating with embedded systems and the Internet of Things [33,34,35,36]. The performance comparison with the existing investigations using the Physionet Apnea-ECG dataset was presented in Table 5. It can be observed that some related work outperformed the proposed approach, such as [10,12,15,19]. However, the proposed method used the complete ECG data segments to increase the overall accuracy. The relative weight of each segment was computed to filter each ECG segment based on its quality instead of dropping them [10]. In addition, the proposed method did not include any estimation of the ECG characteristics, such as the QRS complex, to reduce the probability of incorrect classification. The estimation of the QRS complex may lead to inaccurate findings if the algorithm is inadequate or if the signal is noisy [12]. The proposed approach outperformed some of the deep-learning methods, such as CNN as [9,18]. The Gabor spectrogram improved the significance of the deep learning model when applied. However, deep learning methods usually suffer from the high computational cost of training. The proposed approach outperformed the remaining techniques with a significant difference.

## 5. Conclusions

This paper proposed a novel approach for the automated classification and detection of OSA using single-lead ECG signals. This approach used wavelet scattering transformation for signal decomposition into smaller segments. A set of high-order statistics and entropy-based features were calculated from the obtained scattering coefficients to obtain the hidden information patterns. A Random forest classifier was applied to the obtained features to classify the OSA. The classification performance was performed using accuracy, sensitivity, specificity, AUC, F1 measure, and the Kappa coefficient. The experiment results reported a classification accuracy of 91.65% for 60 s of ECG segments on the Physionet Apnea-ECG. The proposed approach provided better performance measures than most of the existing methodologies. In future work, we aim to improve this work by integrating it with zero-shot learning and training the model on different datasets.

## Figures and Tables

**Table 1:** The proposed method’s average performance measure (values %) using different classifiers with hold-out (50-50) cross-validation.

Classifier	ACC	SEN	SPE	Precision	F1	Kappa
AdaBoost	78.43±0.13	69.85±0.31	83.80±0.16	72.97±0.27	71.38±0.20	0.54
ExtraTrees	90.39±0.11	88.16±0.27	91.78±0.18	87.02±0.24	87.59±0.15	0.79
GaussianNB	67.01±0.46	57.69±3.07	72.82±2.59	57.10±1.01	57.33±1.09	0.30
KNN	87.56±0.13	83.10±0.27	90.35±0.18	84.31±0.29	83.70±0.18	0.73
LDA	72.75±0.13	53.56±0.45	84.77±0.30	68.76±0.36	60.21±0.21	0.39
LR	73.52±0.10	56.14±0.42	84.31±0.28	68.98±0.36	61.90±0.19	0.41
QDA	70.80±6.01	71.15±9.91	70.61±15.71	62.10±6.99	65.39±1.65	0.41
RF	90.23±0.12	87.42±0.29	91.99±0.16	87.24±0.26	87.33±0.17	0.79
SGD	73.36±0.63	59.05±3.03	82.28±2.05	67.60±1.84	62.94±1.38	0.42
SVM	84.14±0.26	76.88±0.77	88.69±0.15	80.99±0.20	78.88±0.43	0.66
XgBoost	89.16±0.12	86.46±0.30	90.84±0.17	85.49±0.22	85.97±0.16	0.77

**Table 2:** The proposed method’s average performance measure (values %) using different classifiers with 10-fold cross-validation.

Classifier	ACC	SEN	SPE	Precision	F1	Kappa
AdaBoost	79.43±0.28	70.47±0.11	83.77±0.07	73.08±0.08	71.74±0.07	0.55
ExtraTrees	91.44±0.13	89.09±0.07	92.24±0.04	87.76±0.06	88.42±0.04	0.81
GaussianNB	68.17±0.22	54.23±0.16	75.71±0.09	58.31±0.05	56.13±0.08	0.30
KNN	88.49±0.12	83.61±0.04	90.88±0.04	85.14±0.06	84.37±0.03	0.75
LDA	73.48±0.19	53.74±0.02	84.67±0.01	68.66±0.02	60.28±0.02	0.40
LR	74.09±0.29	55.91±0.03	84.37±0.01	69.10±0.03	61.81±0.03	0.42
QDA	73.28±0.48	72.37±0.10	71.21±0.12	61.22±0.07	66.26±0.05	0.42
RF	91.51±0.14	88.75±0.08	92.44±0.03	88.00±0.04	88.37±0.04	0.81
SGD	74.15±0.21	58.11±0.98	82.99±0.70	68.20±0.57	62.66±0.39	0.42
SVM	85.65±0.15	79.11±0.03	88.86±0.02	81.61±0.02	80.33±0.02	0.68
XgBoost	90.07±0.19	87.07±0.09	91.17±0.05	86.04±0.07	86.55±0.06	0.78

**Table 3:** The proposed method’s average performance measure (values %) using different classifiers with hold-out cross-validation after applying PCA.

Classifier	ACC	SEN	SPE	Precision	F1	Kappa
AdaBoost	73.16±0.20	59.20±0.46	81.86±0.26	67.05±0.34	62.88±0.32	0.42
ExtraTrees	86.54±0.11	80.97±0.25	90.02±0.18	83.53±0.26	82.23±0.14	0.71
GaussianNB	69.01±1.37	40.01±9.72	87.14±4.36	66.43±2.08	49.21±6.84	0.29±0.05
KNN	86.07±0.12	80.44±0.34	89.58±0.25	82.81±0.39	81.61±0.15	0.70
LDA	71.23±0.19	51.23±0.65	83.76±0.30	66.39±0.40	57.83±0.37	0.37
LR	72.33±0.17	54.55±0.33	83.42±0.36	67.24±0.41	60.23±0.16	0.39
QDA	69.45±3.20	46.88±21.35	83.56±14.85	68.85±8.56	51.71±13.34	0.32±0.08
RF	86.83±0.12	81.78±0.41	89.98±0.20	83.57±0.27	82.66±0.16	0.72
SGD	72.08±0.88	55.78±6.97	82.27±3.18	66.46±1.67	60.34±3.97	0.39±0.03
SVM	82.68±0.25	74.74±0.77	87.66±0.19	79.16±0.21	76.88±0.43	0.63±0.01
XgBoost	85.68±0.14	80.76±0.21	88.76±0.30	81.79±0.41	81.27±0.17	0.70

**Table 4:** The proposed method’s average performance measure (values %) using different classifiers with 10-fold cross-validation after applying PCA.

Classifier	ACC	SEN	SPE	Precision	F1	Kappa
AdaBoost	73.87±0.23	58.89±0.20	82.24±0.13	67.46±0.12	62.88±0.12	0.42
ExtraTrees	87.38±0.21	81.48±0.11	90.19±0.03	83.84±0.05	82.64±0.08	0.72
GaussianNB	70.65±0.55	39.81±0.18	87.62±0.33	66.91±0.30	49.63±0.17	0.30
KNN	87.04±0.14	81.16±0.09	90.02±0.05	83.56±0.08	82.34±0.07	0.72
LDA	71.89±0.15	51.22±0.02	83.84±0.01	66.46±0.01	57.85±0.02	0.37
LR	73.00±0.19	54.41±0.02	83.53±0.02	67.37±0.03	60.20±0.02	0.39
QDA	72.96±0.21	51.60±0.28	85.06±0.26	68.66±0.19	58.62±0.29	0.38
RF	88.00±0.16	82.90±0.07	90.36±0.04	84.31±0.06	83.59±0.05	0.73
SGD	73.25±0.23	56.61±1.22	82.12±0.78	66.56±0.55	61.03±0.55	0.40
SVM	84.05±0.19	76.48±0.03	87.56±0.01	79.35±0.02	77.89±0.02	0.64
XgBoost	86.71±0.21	81.52±0.10	88.99±0.04	82.23±0.05	81.87±0.05	0.71

**Table 5:** Comparison of the performance measures between the proposed method and previous works.

Methodology	ACC	SEN	SPE
QRS + RR intervals [10]	96.5	92.9	100
QRS [11]	80.5	-	-
QRS + Reconstructed Phase Space [12]	94.8	94.16	95.42
RR + extreme learning machine [13]	82.5	81.9	82.8
dual-tree complex wavelet transform [14]	84.4	90.38	74.84
TQWT + Statistical Features [37]	88.88	87.58	91.49
DWT + Non-linear features [15]	92.98	91.74	93.75
QRS [38]	84.74	84.71	84.69
RQA + HRV [39]	85.26	86.37	83.37
TQWT + normal inverse Gaussian [40]	87.33	81.99	90.72
statistical and spectral features [41]	85.97	84.14	86.83
Empirical Mode Decomposition [42]	83.77	85.2	82.79
wavelet filter banks + non-linear [43]	90.87	92.43	88.33
HRV + Non-linear features [44]	77.27	79.25	75.32
QRS [45]	82.12	88.41	72.29
Bandpass Filtering + CNN [9]	87.9	81.1	92
RRI + CNN [18]	85.58	-	88.26
deep learning model [19]	94.81	-	-
HRV + Ensemble Learning [46]	84.61	84.78	84.44
WST + Entropy-based Features	91.65	88.83	92.47
